# Characterizing, controlling and eliminating residual malaria transmission

**DOI:** 10.1186/1475-2875-13-330

**Published:** 2014-08-23

**Authors:** Gerry F Killeen

**Affiliations:** Ifakara Health Institute, Environmental Health and Ecological Sciences Thematic Group, Ifakara, Morogoro United Republic of Tanzania; Liverpool School of Tropical Medicine, Vector Biology Department, Pembroke Place, Liverpool, UK

**Keywords:** Malaria, Residual transmission, Vector control, *Anopheles*, Mosquito, Elimination

## Abstract

Long-lasting insecticidal nets (LLINs) and indoor residual spraying (IRS) interventions can reduce malaria transmission by targeting mosquitoes when they feed upon sleeping humans and/or rest inside houses, livestock shelters or other man-made structures. However, many malaria vector species can maintain robust transmission, despite high coverage of LLINs/IRS containing insecticides to which they are physiologically fully susceptible, because they exhibit one or more behaviours that define the biological limits of achievable impact with these interventions: (1) Natural or insecticide-induced avoidance of contact with treated surfaces within houses and early exit from them, thus minimizing exposure hazard of vectors which feed indoors upon humans; (2) Feeding upon humans when they are active and unprotected outdoors, thereby attenuating personal protection and any consequent community-wide suppression of transmission; (3) Feeding upon animals, thus minimizing contact with insecticides targeted at humans or houses; (4) Resting outdoors, away from insecticide-treated surfaces of nets, walls and roofs. Residual malaria transmission is, therefore, defined as all forms of transmission that can persist after achieving full universal coverage with effective LLINs and/or IRS containing active ingredients to which local vector populations are fully susceptible. Residual transmission is sufficiently intense across most of the tropics to render malaria elimination infeasible without new or improved vector control methods. Many novel or improved vector control strategies to address residual transmission are emerging that either: (1) Enhance control of adult vectors that enter houses to feed and/or rest by killing, repelling or excluding them; (2) Kill or repel adult mosquitoes when they attack people outdoors; (3) Kill adult mosquitoes when they attack livestock; (4) Kill adult mosquitoes when they feed upon sugar or; (5) Kill immature mosquitoes in aquatic habitats. To date, none of these options has sufficient supporting evidence to justify full-scale programmatic implementation. Concerted investment in their rigorous selection, development and evaluation is required over the coming decade to enable control and, ultimately, elimination of residual malaria transmission. In the meantime, national programmes may assess options for addressing residual transmission under programmatic conditions through pilot studies with strong monitoring, evaluation and operational research components, similar to the Onchocerciasis Control Programme.

## Background

Although hundreds of *Anopheles* species have been described worldwide, certain biological and environmental factors distinguish a small subset of a few dozen that actually mediate transmission of *Plasmodium* parasites to humans in the wild [1, 2]. First, only a subset of *Anopheles* species are *physiologically competent* vectors, meaning that they can support parasite development all the way from gametocytes through to sporozoites that are infectious to humans, even if that only occurs under artificial experimental conditions [3]. Second, a physiologically competent vector can only transmit malaria outside a laboratory if it actually bites humans and survives long enough in the wild for sporogonic development of parasites to be completed [2–4]. The survival and reproduction of mosquitoes, as well as sporogonic development of parasites within their bodies, are both heavily dependent upon temperature, humidity and rainfall, so malaria transmission is most widespread and intense in the warmer, wetter regions of the tropics [5, 6]. However, the specific behaviours exhibited by each distinct vector population in a given locality are not only important determinants of their *vectorial capacity* to mediate malaria transmission [1, 2, 7] but also their vulnerability to control or even elimination [8–13].

### Feeding upon humans as a driver of malaria transmission and intervention impact

*Plasmodium vivax* and *Plasmodium falciparum* account for the vast majority (>90%) of human malaria infections worldwide and both can only be transmitted from one human to another via mosquitoes [5, 6]. The entire infectious reservoir for these hugely important pathogens is to be found exclusively in humans, so their transmission requires that a mosquito must bite at least two people in its lifetime. Sustained local transmission therefore requires that local *Anopheles* mosquitoes are not only physiologically competent and survive long enough for complete sporogonic development of malaria parasites within their bodies, but also that they feed at least occasionally upon humans.

**Figure 1:**
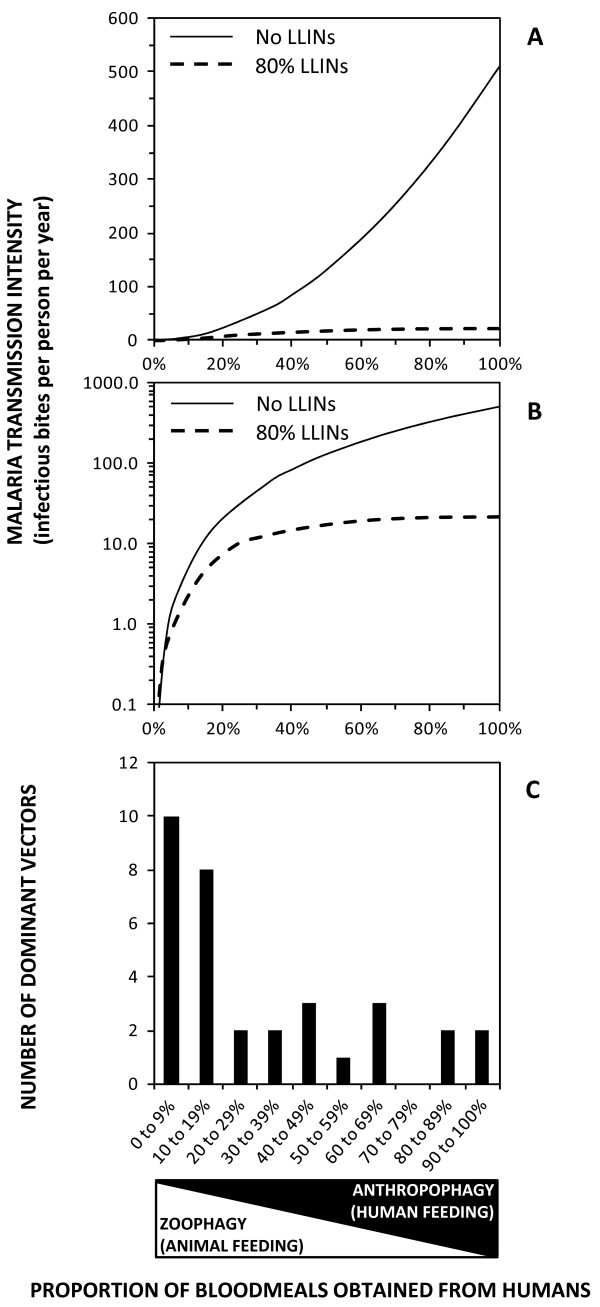
**The importance of feeding upon humans as a determinant of malaria transmission and vector control impact. A** and **B**: Simulated relationship between malaria transmission intensity mediated by an *Anopheles* mosquito population and the proportion of blood meals that these vectors obtain from humans, in the presence and absence of long-lasting insecticidal nets (LLINs) with a mean nightly usage rate of 80%, presented with a linear **(A)** and logarithmic **(B)** vertical axis (Adapted from reference 11). **C**: Frequency distribution for the mean proportion of blood meals obtained from humans for the 33 most important locally dominant malaria vectors worldwide as reviewed in reference [7].

**Figure 2:**
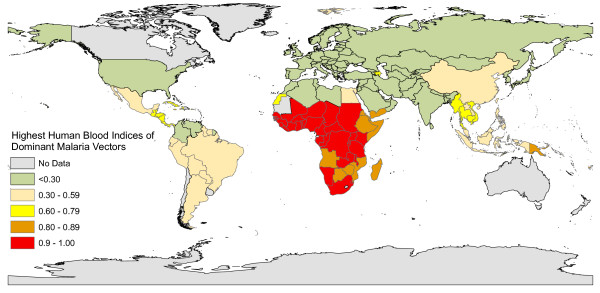
**Global map of the highest human blood index among nationally important vectors, as extracted from reference 7 and kindly drafted by Fredros Okumu and Alex Limwagu.**

Malaria transmission intensity can be measured as the *entomological inoculation rate* (EIR), expressed as the number of times per year an individual human resident is bitten by mosquitoes with infectious sporozoites in their salivary glands. EIR increases approximately exponentially as the proportion of blood meals that a vector population obtains from humans increases (Figures 1A and 1B), so the distribution of exceptionally high transmission intensities across equatorial Africa and some parts of the Pacific may be readily explained by the presence of *Anopheles* species that rely almost exclusively upon humans for blood (Figure 2). However, it is also critical to note that where a vector is heavily reliant upon human blood, it will often be consequently vulnerable to population control with indoor residual spray (IRS) or long-lasting insecticidal net (LLIN) products designed to kill mosquitoes when they encounter people or houses (Figures 1A and 1B) [10–15].

### Targeting stereotypical indoor resting and feeding behaviours of human-specialized *Anopheles*

Given the importance of feeding upon humans as a determinant of malaria transmission (Figures 1 and 2), it is unsurprising that the most *anthropophagic* (predominantly feed upon humans) vectors are by far the best studied. It is also understandable that the specialized behavioural adaptations, which many of them have in common, have dominated thinking about malaria transmission and vector control for decades. Many of the most regionally important vectors of malaria, like *Anopheles gambiae*, *Anopheles arabiensis* and *Anopheles funestus* from Africa [16], *Anopheles stephensi, Anopheles culicifacies* and *Anopheles punctulatus* from Asia [17, 18], or *Anopheles darlingi*, *Anopheles punctimacula*, *Anopheles nunetzovari* (species B or C) and *Anopheles albimanus* from Latin America [19, 20] prefer to feed in the middle of the night when most humans are typically asleep, immobile and vulnerable to attack. Feeding indoors at night is, therefore, the behaviour that is targeted by use of LLINs to protect sleeping humans.

Indoor feeding is then often conveniently followed by resting within the same sheltered domestic structure for one or two nights while the blood meal is digested and eggs are developed. Applications of insecticides to houses by IRS, to kill mosquitoes resting on inner surfaces of the walls and roof after they have fed upon the human occupants, is therefore a highly effective strategy for controlling populations of vectors that rest indoors as a matter of preference.

**Figure 3:**
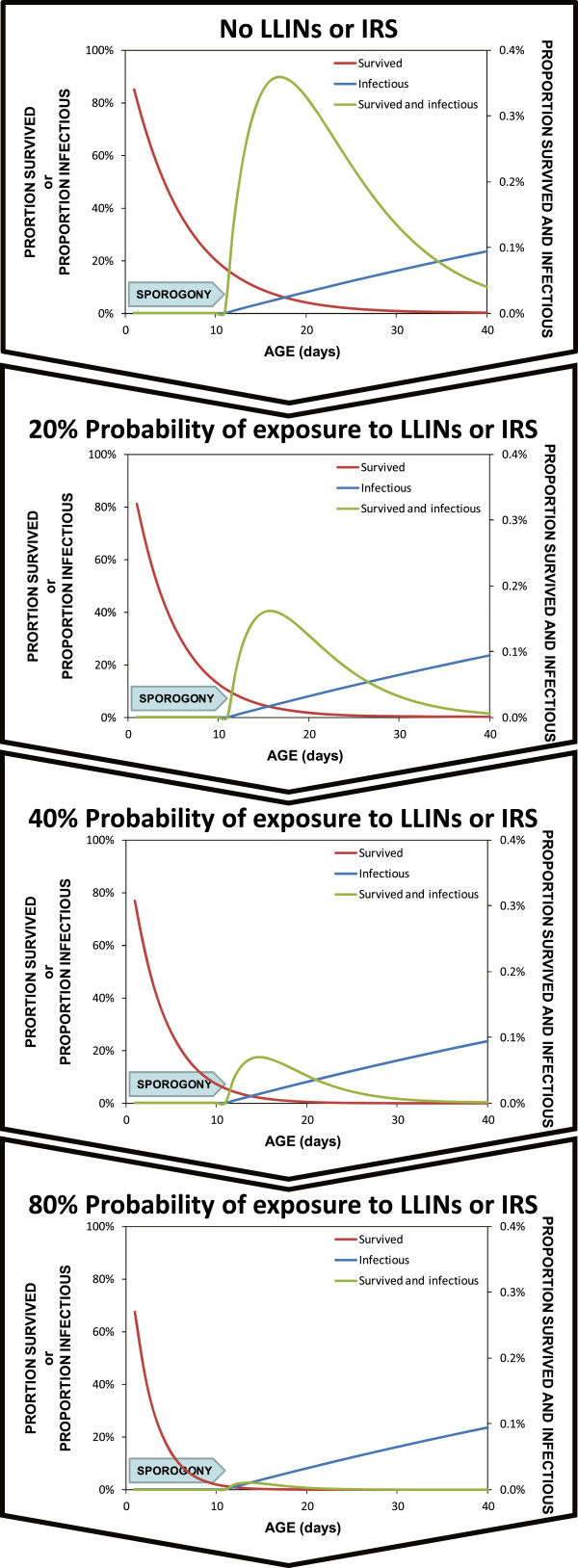
**Progressive dramatic reduction of mosquito survival and infection probability as an increasing proportion of available blood meals are covered with LLINs or IRS.** The probability curves presented represent the outputs of simulations implemented exactly as previously described [14] at 0, 20, 40 and 60% biological coverage of all available blood resources [10, 13] with LLINs that kill 60% of all mosquitoes encountering them.

The success of LLINs and IRS in combating malaria transmission by stereotypical vectors which feed and rest indoors is well established [21, 22]. Even imperfect coverage of entire human populations with LLINs and IRS can have massive benefits for all members of malaria-afflicted communities, including those whose houses and sleeping spaces are not directly protected [23]. This community-wide *mass effect* occurs because those who are directly protected actually kill mosquitoes when they attempt to feed, so that vector survival rates and population densities are reduced, resulting in far fewer mosquitoes living long enough to mediate transmission between humans (Figure 3) [23]. Furthermore, LLINs and IRS can have a surprisingly dramatic impact on overall population size of stereotypical vectors that depend heavily upon feeding on humans and resting inside houses [13]: Entire vector populations may be eliminated, or at least negated as a cause of malaria, including documented examples for *An. gambiae* and *An. funestus* in Africa, *An. darling* and *An. nuneztovari* in Latin America, and *An. punctulatus* as well as *Anopheles koliensis* in the Pacific [13].

### Persistence of residual transmission after scale up of LLINs and IRS: failure or limitation?

**Figure 4:**
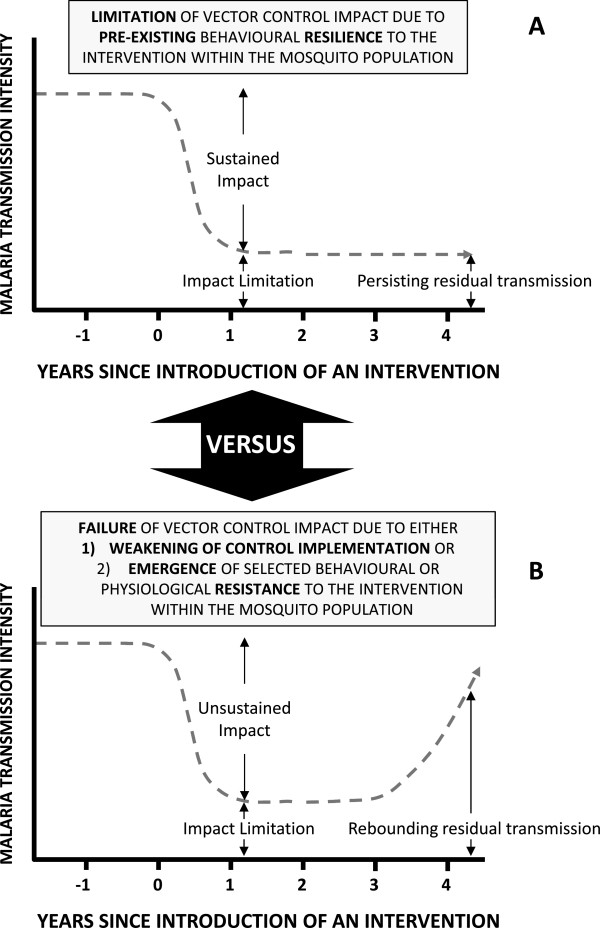
**A schematic illustration of the differing trajectories of impact of an intervention upon malaria transmission by a vector population under the distinctive scenarios of either (A) Stable limitation of sustained impact arising from expression of pre-existing behavioural traits within a resilient vector population, or (B) Failure of impact and resurgence of malaria transmission when, either intervention programme implementation quality and coverage weakens, or selected behavioural or physiological traits emerge within an increasingly resistant, rebounding vector population** [31] **.**

Despite the impressive successes that have been achieved by targeting stereotypical vectors that feed and rest indoors with IRS and LLINs, complete elimination of malaria has rarely been achieved outside of areas that had marginal transmission levels to begin with [13, 24, 25]. There are fundamental limits to how much impact even the best implemented LLIN or IRS programmes can achieve in most tropical settings (Figure 4A) and it is essential to recognize that this phenomenon is normal and has been repeatedly reported from a variety of settings over the course of the last half century [13, 26–30]. It is crucial to distinguish between such fundamental *limitations* of a given vector control strategy that has incomplete but nevertheless valuable and stable levels of impact that may be sustained over the long term (Figure 4A), and a genuine *failure* of an intervention programme that enables the vector population and malaria transmission to *rebound* (Figure 4B) [28, 29, 31].

While rebounding vector populations and malaria transmission intensities have been most commonly associated with failures of implementation and funding for vector control programmes, the emergence of physiological resistance to insecticides within these recovering mosquito populations has also been implicated [32]. Physiological resistance of mosquitoes to insecticides, resistance of policy makers to funding support, and resistance of the general public to interventions, are all widely accepted as important causes of vector control programme failure and have been reviewed in detail elsewhere [33]. However, it is less commonly understood that the fundamental limits of what can be achieved with IRS, LLINs, or indeed any other vector control strategy, are primarily defined by the behavioural traits of mosquitoes [13, 19, 26–30, 34–36].

It is also extremely important to understand that many of the behavioural traits which allow residual populations of vector mosquitoes to survive and persistently transmit malaria despite high coverage of LLINs or IRS, appear to have always been present in these populations [13, 26–29]. As such, they are better described as pre-existing behavioural *resilience* (Figure 4A), rather than emerging *resistance* in the strict sense (Figure 4B) [28, 29]. While the possibility that true behavioural resistance may emerge in response to intervention pressure upon vector populations cannot be ignored [30, 37, 38], no clear-cut instance of this phenomenon has been documented in the field [26, 28]. While many instances of apparently altered or atypical patterns of mosquito behaviour have been reported, and some of these are difficult to explain without assuming the emergence of behavioural resistance [38], it is technically difficult to unambiguously attribute these to the emergence of heritably altered preference traits. Instead, these observations of altered mosquito behaviours may well arise instead from either (1) altered taxonomic composition of the vector population due to differential suppression of various species and sub-species taxa as a result of their varying degree of behavioural suitability to control with IRS or LLINs, or (2) altered expression of innately flexible behaviours by mosquitoes in response to the altered patterns of blood and resting site resource availability in their environment following IRS or LLIN scale up [26, 28]. However, regardless of whether these behaviours reflect the selection of new heritable resistance traits, the altered expression of pre-existing resilience traits, or a combination of both, the fact remains that they will have to be deliberately and specifically targeted with new vector control tools to achieve malaria elimination [37, 38].

### Beyond the stereotype: Mosquito behaviours that limit the impact of LLINs and IRS

Unfortunately, the small number vectors that feed predominantly on humans are responsible for such a disproportionately large share of the world’s malaria burden (Figures 1 and 2) that their specific behavioural adaptations, to attacking sleeping humans inside houses and then resting there, have been widely and inaccurately stereotyped as typical of malaria vectors in general. However, *Anopheles* species that exhibit high vectorial capacity, but also high susceptibility to control or even elimination with LLINs and IRS, are the exceptions rather than the rule among malaria vectors. Broadly speaking, known behaviours that buffer mosquito populations and malaria transmission against IRS and LLINs fall into two main categories: 1) Insecticide contact avoidance and early-exit behaviours that minimize the exposure hazard faced by vectors that preferentially feed indoors, and, 2) Animal-feeding and outdoor-feeding behavioural preferences that allow mosquitoes to minimize contact with insecticides targeted at humans and houses altogether.

#### Insecticide avoidance and early-exit behaviours among indoor-feeding vectors

**Figure 5:**
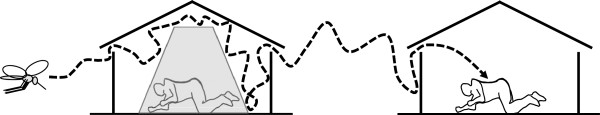
**A schematic illustration of how mosquitoes may survive despite high coverage of long-lasting insecticidal nets or indoor residual spraying by entering, but then rapidly leaving houses protected with LLINs or IRS without exposing themselves to lethal doses of the active ingredients, and then continuing to forage until an unprotected blood host is found** [38–41] **.**

Several vector species appear to have always exhibited a pre-existing tendency to exit houses soon after entering, and this represents an important form of behavioural resilience that receives inadequate attention [19, 39–41]. However, it has long been known that such insecticide-avoidance behaviours can also be induced or exacerbated by irritant or repellent active ingredients that can enhance personal protection afforded by a protective measure but may also undermine potentially greater impact upon vector populations that normally feed indoors upon humans [42–44]. Recent simulation analyses suggest that expression of such avoidance behaviours, which allow vectors to either avoid, or enter but then safely leave, houses protected by LLINs and/or IRS, actually represent an optimal survival strategy for mosquitoes because it allows them to maximize their feeding probability by continuing to forage until they find unprotected hosts [38] (Figure 5). It is therefore understandable that some remarkably robust populations of *An. arabiensis* [45], a species known to be capable of avoiding insecticide exposure when they enter houses [39–41], have retained their historically strong preferences for feeding indoors despite exposure to high levels of LLIN usage by humans for over a decade [46].

**Vectors that enter but then rapidly exit from human habitations** Several important vector species around the world, such as *An. arabiensis* from Africa [39–41] or *An. darlingi*, *An. punctimacula* and *An. nunetzovari* (species B or C) from Latin America [19], enter houses but then rapidly exit again, regardless of whether or not they have successfully fed upon the human occupants. Even when such vectors make direct contact with an insecticide-treated wall [19] or blood host [47], they rarely do so for longer than one or two minutes so that fatal exposure is avoided. Interestingly, this particular combination of behaviours was considered the most important obstacle to elimination of malaria from the Americas with IRS during the Global Malaria Eradication Programme [19] and the same is probably true in many parts of east Africa today where *An. arabiensis* [39–41] is often responsible for most of the persisting residual transmission following successful scale up of LLINs [45, 48–50].

**Induced vector avoidance of contact with repellent or irritant insecticides** It has long been known that even mosquitoes which are normally very susceptible to control with LLINs or IRS, due to the fact that they usually feed and rest indoors, may choose to curtail or avoid periods of physical contact with insecticides if they can detect them with their sensory organs [42–44]. Such stimulant insecticides artificially induce or exacerbate early exit behaviours, ultimately attenuating mosquito exposure to lethal doses [14, 15, 42–44, 51]. Behaviour-modifying insecticides which require direct physical contact with a mosquito to induce an avoidance response are known as contact irritants, while those that the mosquito can sense in the air at a distance from the treated surface, and then choose to avoid contact with, are known as spatial repellents [51, 52]. Many vector mosquito species may feed and rest indoors in the absence of LLINs or IRS with such irritant or repellent insecticides, but the presence of these active ingredients may induce them to leave houses prematurely or even avoid entering in the first place [26, 43, 51, 53].

While many manufacturers emphasize that their products combine behaviour-modifying repellent and irritant properties with contact toxicity, this ignores the fact that these three actions occur sequentially and competitively in that order [14, 15, 51]. No individual mosquito approaching a protected human can be classified as having been affected by two or more of these actions: By definition, an insecticide can only kill a mosquito if it is not first irritated upon contact, and neither of these outcomes is possible if it is repelled before making contact [51, 54]. A given LLIN or IRS product may be optimized to maximize the irritant and repellent actions of sub-toxic doses of the active ingredient, thereby increasing the level of personal protection that is most important for preventing transmission by mosquitoes that only feed occasionally upon humans [10, 11], especially if they do so outdoors [8, 10, 15]. However, this choice will reduce exposure of mosquitoes to toxic doses of the active ingredient that can kill them outright and therefore undermines the massive community-level protection that can be achieved through vector population suppression where vectors are heavily dependent upon human blood for their survival [8, 10, 14, 15, 51, 55]. In summary, these alternative modes of action must be traded off against each other: While a contact toxin may have no advantage over a behaviour-modifying irritant or repellent where local vectors populations are not dependent on human blood for their survival [8, 10, 11], in situations where vectors predominantly feed upon people indoors and can be killed inside houses by toxic insecticides delivered as LLINs or IRS, supplementing these with any repellent or irritant action may ultimately undermine their potential to control [8, 10, 14, 15, 51, 55] or even eliminate [13] such stereotypically synanthropic vectors.

It is particularly notable that the principles underlying the necessity to choose between toxic *versus* irritant and repellent modes of action were widely accepted during the era of the Global Malaria Eradication Programme (GMEP) [42–44]. Indeed, by the end of the GMEP, it was already recognized that implementing IRS with DDT, which is known to be both repellent and irritant [54, 56], generally had less impact than implementing IRS with insecticides that lacked these properties [44]. In fact, the main reasons why DDT was often selected as the active ingredient of choice were its affordability and longer duration of residual activity [44]. It is therefore timely to note that these principles were played out again in recent experimental hut trials in which IRS with DDT slightly attenuated the toxic effects of one pyrethroid-treated net product when the two were combined in the same hut [40, 41]. It is also noteworthy that the *An. arabiensis* population that these IRS-LLIN combinations were evaluated against already exhibited early exiting behaviour even in the absence of insecticides [40, 41]. While new vapour-phase repellents to prevent transmission exposure outdoors are clearly essential, they should be applied cautiously inside houses wherever indoor-feeding or indoor-resting mosquitoes with strong preferences for human blood and high vectorial capacity exist [8, 10, 14, 15, 51]. In such circumstances, purely toxic insecticide formulations delivered to houses, possibly in the form of IRS and LLINs, are likely to achieve greater impact [8, 10, 14, 15, 51].

#### Behavioural preferences for feeding outdoors and upon animals

**Figure 6:**
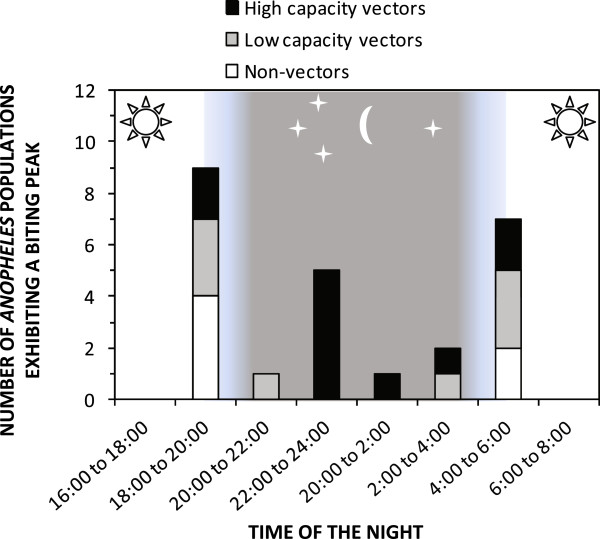
**Frequency distribution of the preferred biting times for 25 separate populations of 11 Latin American** ***Anopheles*** **species, which were classified as either: 1) potent primary vectors; 2) weak, incidental or secondary vectors; or 3) non-vectors (Adapted from reference** [19] **).**

The majority of malaria vector species worldwide can be described as *zoophagic* because they actually feed predominantly upon animals (Figures 1C and 2). Since they rarely bite humans, they are correspondingly less efficient at transmitting malaria (Figures 1A and 1B). However, these less potent vectors are often difficult to control with LLINs or IRS, not only because they usually feed upon unprotected animals (Figures 1A and 1B), but also because, they usually prefer to feed at dusk and dawn (Figure 6) when most of their human victims are outdoors, beyond the protective reach of these prevention measures. Zoophagic mosquitoes with moderate vectorial capacity, most of which primarily feed and rest outdoors, are widespread throughout the tropics (Figures 1C and 2). These species often respond poorly to LLIN or IRS interventions because the technologies are designed to target the stereotypical behaviours of the smaller number of more potent, human-specialized species (Figure 1) that mediate most, but by no means all, of the global malaria burden (Figure 2).

**Vectors that feed upon animals** Many mosquitoes are highly specialized in terms of their preferred blood sources [57], and exhibit enormous diversity of preference even between morphologically identical members of a single species complex [58–61]. Humans represent only one of the many vertebrates *Anopheles* have adapted to exploit [57] and the vast majority of malaria vectors feed primarily on animals (Figures 1C and 2). EIR levels as low as 0.1 infectious bites per person per year are typically sufficient to sustain endemic populations of malaria parasites [62–64] so a mosquito species may be capable of sustaining endemic malaria transmission even if it feeds only rarely upon humans (Figure 1A and B). So while a small proportion of the world’s overall malaria burden is caused by *Anopheles* which prefer to feed upon animals, these comprise the majority of all malaria vectors worldwide (Figures 1C) and affect the majority of the global at-risk human population who live outside of Oceania or Africa south of the Sahara [5, 6] (Figure 2).

Most of the world’s at-risk population [5, 6] is therefore exposed to limited, but nevertheless self-sustaining, malaria transmission by mosquitoes which have moderate vectorial capacity since potentially infectious humans contribute only a minority of the blood meals they rely upon to survive and reproduce (Figure 2). While the malaria transmission caused by this near-ubiquitous plethora of zoophagic vectors occurs at relatively modest intensities, it is also relatively unresponsive to control with measures that target human blood sources, such as IRS and LLINs (Figure 1A and B). While high coverage of these measures can achieve useful community-wide reductions of malaria transmission by preventing human-vector contact [10, 11], the actual impact upon population density and survival of vectors is likely to be negligible given that these mosquitoes obtain most of the blood they need from animals (Figure 1A and 1B). This disconnect, between targeting IRS and LLINs at the humans who need to be protected and not at the animals that mosquitoes depend on for survival, creates a gap in protective coverage of the blood resources that actually sustain the vector population [8, 10, 11, 13]. High coverage of all these blood resources with interventions that render them hazardous to mosquitoes is required if population control is to be achieved, rather than merely direct personal protection of the subset that humans represent [8, 10, 11, 13].

**Figure 7:**
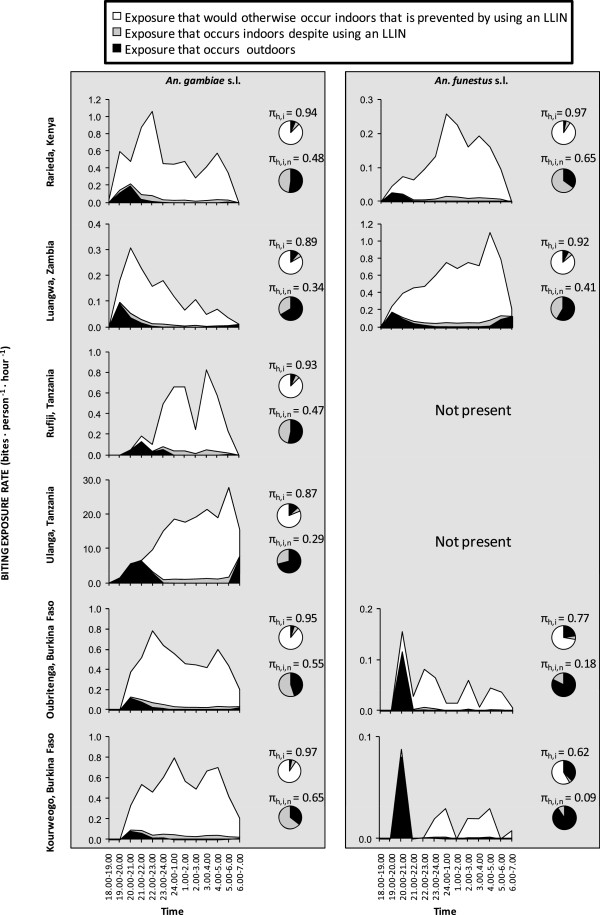
**Estimates of the proportion of human exposure to African malaria vector populations that occurs indoors for both unprotected residents (** ***π***_***h,i***_ **) and users of long-lasting insecticidal nets (** ***π***_***h,i,n***_ **), from field sites across eastern, southern and western Africa**[16]**, as previously calculated** [71, 72] **and presented in summary form** [29]**.** Original data kindly provided by Bernadette Huho, Olivier Briët, Aklilu Seyoum, Chadwick Sikaala, Nabie Bayoh, John Gimnig, Fredros Okumu, Diadier Diallo, Salim Abdulla and Tom Smith.

**Vectors that feed on people when they are active outdoors** Even amongst the stereotypically nocturnal major vectors of Africa, which overwhelmingly prefer to feed at night when people are asleep [16], a small but important portion of feeding activity does occur at dawn and dusk. While this represented a minor fraction of historical malaria transmission in unprotected African populations, it now typically accounts for approximately half of all transmission exposure to residual vector populations for individuals protected against most indoor exposure by LLINs [29] (Figure 7). Furthermore, several recent reports of atypical or altered biting patterns by these same vector species suggest that higher proportions of transmission now occur outdoors in the evenings and early mornings [50, 65–70] so the majority of residual exposure of net users most probably occurs outdoors in many African settings.

**Figure 8:**
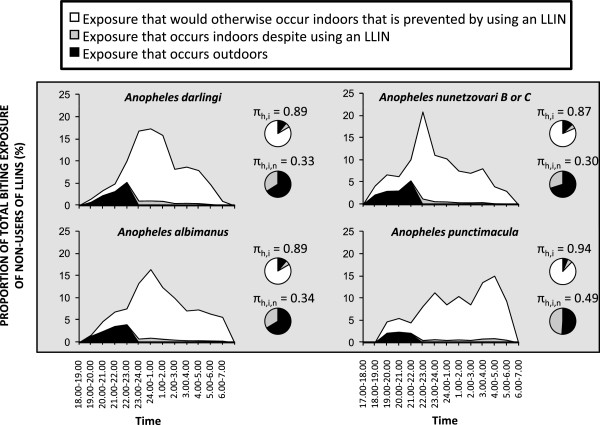
**Historical estimates of the proportion of human exposure to Latin American malaria vector populations in Colombia that would have occured indoors for both unprotected residents (** ***π***_***h,i***_ **) and users of modern long-lasting insecticidal nets (** ***π***_***h,i,n***_ **), calculated as originally described** [19, 20] **, except for the breakdown of indoor exposure into the fractions that would and would not be prevented by net use** [71, 72] **.**

Beyond Africa, the four major Latin American vectors which were historically implicated in the failure of IRS to eliminate malaria from Colombia [19, 20] predominantly fed upon humans when they slept indoors at night (Figure 8), to essentially the same extent as stereotypical African vectors (Figure 7). However, in this historical example, at least half of residual transmission by all four of these Latin American species would also have occurred outdoors if all residents had used a modern LLIN (Figure 8).

**Figure 9:**
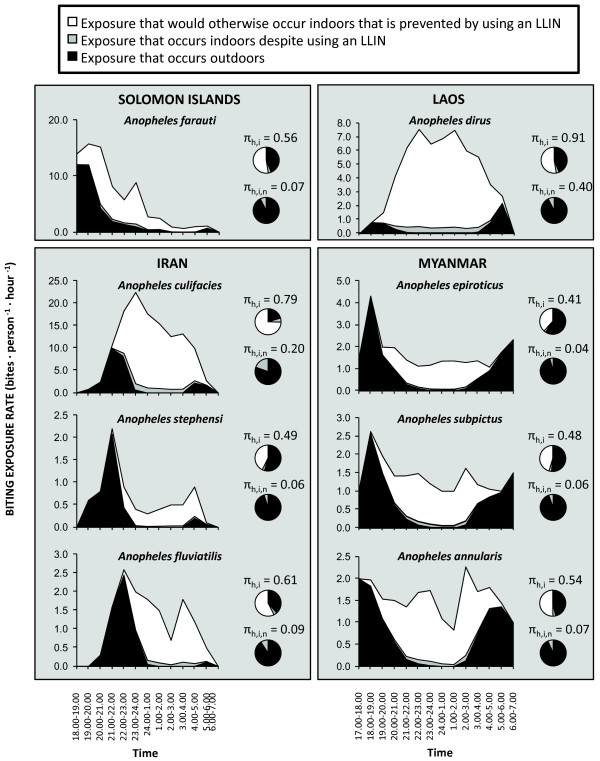
**Estimates of the proportion of human exposure to Asian malaria vector populations that occurs indoors for both unprotected residents (** ***π***_***h,i***_ **) and users of long-lasting insecticidal nets (** ***π***_***h,i,n***_ **), from the Solomon Islands** [75]**, Laos** [76]**, Iran** [17] **and Myanmar** [77, 78]**, calculated as previously described** [71, 72]**, except that in the Iranian examples, indoor and outdoor biting densities were assumed to be equal because they were not reported separately** [17]**.** Original data from the Solomon Islands and Myanmar were kindly provided by Hugo Bugoro, Tanya Russell, Frank Smithuis and Nick White.

While *Anopheles dirus* in south-east Asia can exhibit similarly stereotypical nocturnal, indoor-feeding behaviour, this is unusual amongst vectors within the region (Figure 9) and there are numerous other examples of *An. dirus* mostly feeding outdoors, much earlier in the evening [73]. Similarly to their African counterparts, at least half of the exposure of residents who use LLINs to such stereotypically nocturnal, indoor-feeding vectors populations where they occur in south-east Asia occurs while they are outdoors (Figure 9). This is particularly worrying in relation to *An. dirus*, the most important vector in the greater Mekong region where containment of growing parasite resistance to artemisinin-based drugs will most probably require elimination of transmission at sub-regional level [74].

By comparison with these stereotypical anthropophagic *Anopheles* that are all known to be associated with intense transmission of malaria, species with lower vectorial capacity, most of which are more inclined to feed upon animals, typically exhibit no such adaptation to feeding in the middle of the night when people are asleep. Instead, such *crepuscular* vectors feed at dawn and/or dusk, or during the hours of darkness immediately before dawn and after dusk (Figure 6) when most people are awake and active, and it is impractical to protect them with LLINs. Feeding upon exposed humans at dawn and dusk predominantly occurs outdoors, and is consequently usually followed by resting outdoors, beyond the reach of IRS.

As just one example of a vector that often departs from the stereotype of indoor feeding and resting, several reports of *An. dirus* feeding outdoors in the evening [73] indicate that the only published example from which exposure distribution could currently be calculated (Figure 9) may not be fully representative of the species in general. Furthermore, remarkably high proportions of malaria transmission occur outdoors across all the other major regions of Asia (Figure 9). In the absence of any preventative measure, approximately half of transmission by all vectors in the south, south-east and Pacific regions of Asia, other than *Anopheles culicifacies*, occurs outdoors so provision of either LLINs or IRS is unlikely to directly protect against this fraction of exposure. Even for *An. culicifacies*, the most endophagic vector on the continent, one fifth of exposure occurs outdoors for non-users of LLINs. Furthermore, for users of LLINs, remaining indoor exposure that nets cannot be expected to completely prevent, accounts for only one fifth of residual transmission by *An. culicifacies*, and less than one tenth of residual transmission by all the other Asian vectors described in Figure 9. It is also notable that apparently altered behaviours, presumably reflecting behavioural resistance in the strict sense [38], have been observed on several occasions following implementation of IRS against these Asian vectors [26, 73]. Clearly, any vector measure selected to complement LLINs as a means of providing direct personal protection must be feasibly applicable by human users outdoors, including during periods when they are active, if it is to confer any meaningful incremental impact.

**Figure 10:**
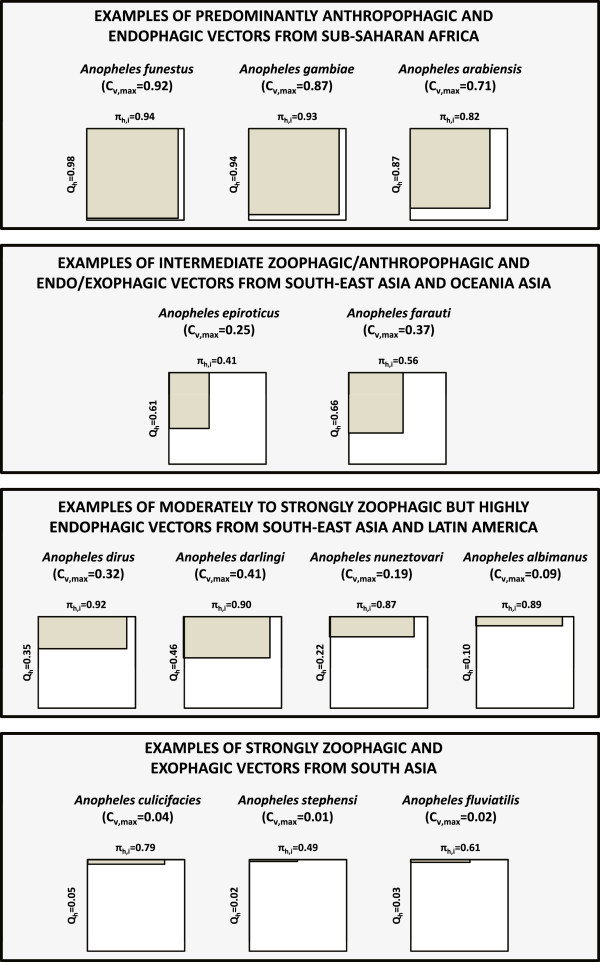
**A graphic illustration of the estimated maximum achievable biological coverage of all blood resources (C**_***v,max***_ **) utilized by the vector species described in Figures** 1 **,** 2 **,** 7 **,** 8 **and** 9 **, for which estimates of both the proportion of blood meals obtained from humans (** ***Q***_***h***_ **) and the proportion of human blood meals obtained indoors (** ***π***_***h,i***_ **) were available.** The width of the grey rectangles relative to that of the white squares represents the limit of personal protection and derived community-wide reduction of mutual human-vector exposure, while their relative area represents the achievable limit of biological coverage of all blood resources that determines the extent to which the density and survival of the vector population can be controlled [8, 10, 11, 13].

The major vectors of south Asia only occasionally feed upon humans [7], and largely do so outdoors [79], so the maximum biological coverage of blood resources that can be achieved with LLINs appears negligible because it is viewed solely in terms of feeding behaviours alone (Figure 10). However, these major vectors are nevertheless remarkably susceptible to control with IRS [22], because they usually rest inside houses and cattle shelters after feeding [79]. This example illustrates just how important it can be to clearly identify and distinguish the specific blood, resting site, sugar, or larval habitat resource, or subsets thereof, that a given intervention actually targets and to quantify the rate at which it is utilized [8, 80]. Such quantitative approaches to surveying mosquito resource utilization behaviour may be useful to distinguish: (1) scenarios in which LLINs or IRS may have little impact, so that alternative vector control strategies are desperately needed; and (2) scenarios such as the south Asian example outlined above, where IRS may be surprisingly effective so additional approaches may be viewed as complementary and secondary, rather than superior, primary alternatives [8, 80].

**Quantifying the limits of biological coverage that are attainable with LLINs and IRS** Achieving population control of malaria vectors, rather than merely personal protection of human individuals and communities, requires that reasonably high mosquito mortality rates are achieved, similar to those depicted at the bottom of Figure 3. However, delivering such a population control impact in practice requires high insecticide coverage of all blood sources, or all associated resting sites, rather than just the fraction represented by the human population [10]. It is, therefore, critical to conceptualize and quantify the influence of feeding upon animals and feeding or resting outdoors in terms of field-measurable, behaviourally-defined gaps in the *de facto* coverage of blood or resting site resources that IRS and LLINs achieve [10, 13]. Indeed the natural limits of what is possible with LLINs, and to a lesser extent IRS, may be represented in terms of gaps in biological coverage of all blood resources [8, 13]. Biological coverage may be plotted as the product of the proportion of blood meals obtained from humans and the proportion of human blood meals obtained indoors, both of which can be readily measured in the field, with the remaining uncovered proportions along each axis of the two-dimensional plot representing the coverage gaps [10, 13]. The data presented in Figures 1, 2, 7, 8 and 9 can therefore be used to illustrate the impressive extent of the biological coverage gaps caused by mosquitoes feeding upon animals, and upon humans when they are outdoors (Figure 10). Even in Africa, where the biological coverage limits of LLINs are generally quite high, it is notable that that the biological coverage gaps for *An. arabiensis* (18%), often the most robust of the three most important vectors in the region, are more than twice as large as those for *An. gambiae* (7%) and *An. funestus* (6%), which have both been eliminated, or almost eliminated, by LLINs or IRS on several occasions [13].

While estimates of biological coverage based on human blood utilization patterns may be relevant to IRS in the many settings in which vectors rest where they feed, they may be misleading where vectors feed indoors but rest outdoors or *vice versa* [8]. Improved entomological survey methods are therefore required to quantify vector utilization of treatable resting site surfaces so that similar, but more directly relevant, biological coverage limits can be estimated for IRS [8, 80].

### The scale of the challenge presented by residual transmission

**Figure 11:**
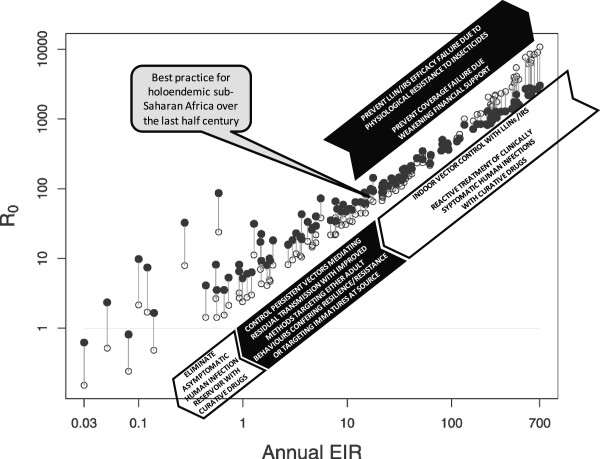
**A schematic representation of the sequential layers of interventions required to eliminate malaria from the most staunchly endemic regions of Africa, adapted from references** [64] **and** [29]**.** White arrows crudely illustrate the impacts of intervention strategies for which reasonable experience and understanding already exists (suppression of high transmission with LLINs or IRS and elimination of sparse residual human parasite reservoirs with drugs). Dark arrows illustrate the potential impact of interventions that urgently need to be developed and evaluated to either maximize impact of existing control measures (adequate and sustainable financing, long-term resistance management) or make more meaningful progress towards elimination (programmatic-scale interruption of residual transmission by behaviourally resilient and/or resistant mosquitoes using novel vector control tools, possibly supplemented with vaccines or chemoprophylaxis).

The best estimates to date all suggest that transmission of *P. falciparum* malaria only drops below self-sustaining levels at EIR values of less than 0.1 infectious bites per year [62–64, 81], so historical values approaching 1000 infectious bites per year reported from several setting are approximately ten thousand times higher than those required to sustain a stable parasite population (R_0_ ≥ 10,000). Thus, even a 99% reduction of transmission by LLINs/IRS would only take control half way along the pathway to elimination and further reductions of similar magnitude would be required to destabilize *P. falciparum* parasite populations in such settings (Figure 11). Residual transmission can therefore be remarkably intense, especially in many parts of Africa and Oceania, where it can occur at intensities far in excess of the thresholds required to be self-sustaining, irrespective of how effectively LLINs, IRS and complementary interventions to diagnose and treat humans are applied [26, 28–30, 35, 36]. Now that history has repeated itself, it must be accepted that these limitations are fundamental and biological in nature, rather than financial or operational [29]. Improved programmatic funding and effectiveness, to deliver better coverage of improved IRS or LLIN products, will not achieve elimination of malaria transmission from most settings because their fundamental limitations of impact are defined by vector behaviours that enable them to avoid fatal contact with these interventions [13, 26–30, 34–36]. Perhaps the most convincing proof of this principle lies in the exceptions: Iran [82] and Sri Lanka [83], for example, are both on the verge of elimination because their southern Asian vectors all predominantly rest indoors [79] and are therefore vulnerable to control with IRS [22], despite feeding largely upon animals [7] and often feeding outdoors on the important occasions when they do attack humans (Figure 9).

### Defining residual malaria transmission

These well-established, fundamental and biological limitations of IRS and LLINs need to be openly and unambiguously acknowledged at all national and international levels of policy and practice. The term *residual malaria transmission* is therefore defined here as all forms of malaria transmission that persist after full universal coverage with effective LLIN and/or IRS interventions has been achieved.

### New and improved vector control options for controlling and eliminating residual malaria transmission

**Figure 12:**
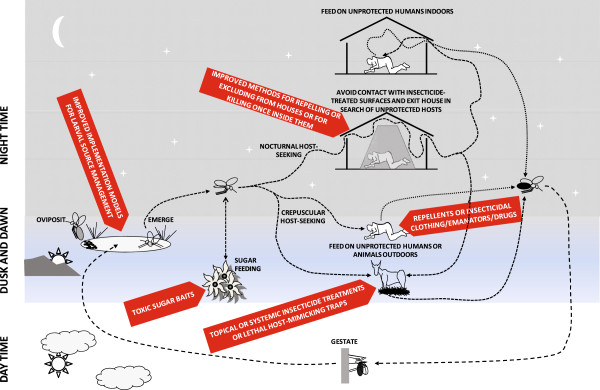
**A schematic summary of how specific behaviours enable mosquito populations to survive and mediate residual malaria transmission despite high coverage of long-lasting insecticidal nets and/or indoor residual sprays, and how these might be tackled with new or improved vector control strategies** [27, 84, 85] **.**

In order to eliminate malaria from most endemic regions of the tropics, concerted investment is required, not only to sustain and consolidate recent gains with LLINs and IRS [33], but also to select, develop and rigorously evaluate supplementary vector control strategies that address residual transmission by deliberately targeting the mosquito behaviours which enable it [8, 26–30, 34–41, 50–55, 67–73, 75, 80]. A very wide diversity of novel or improved strategies for controlling vectors of residual transmission is now emerging (Figure 12).

#### Improving control of house-entering adult mosquitoes

Perhaps the oldest proven means of preventing human exposure to malaria transmission is the modification of houses to prevent mosquitoes from entering them [86] and this time-honoured approach has recently proven successful even in impoverished African settings where housing quality is limited [87–89]. Alternatively, new emanatory products that release vapour-phase insecticides offer the opportunity to repel or kill mosquitoes that would otherwise enter houses and feed upon their occupants [51]. However, the potential for these approaches to interact antagonistically with existing IRS and LLIN interventions needs to be carefully examined [10, 14, 15, 51]. It may also be possible to improve upon the efficacy of IRS and LLIN technologies with enhanced active ingredients and formulations, and insecticidal wall linings are also showing considerable promise [90–92]. However, merely enhancing and refining these conventional indoor control methods may not always address the fundamental behavioural reasons why they achieve little impact upon vectors like *An. arabiensis, An. darlingi*, *An. nunetzovari* (species B or C) and *An. punctimacula* that minimize contact with treated surfaces while resting or feeding indoors [19, 39–41, 47]. Nevertheless, it is encouraging that recent assessments of IRS with a new organophosphate against *An. arabiensis* in east Africa proved far more successful in the absence of LLINs [93] than with them [40, 41], presumably because mosquitoes that have fed are far more inclined to rest in a treated house than those that have not. While exclusively community-level control of malaria transmission by killing mosquitoes after allowing them to feed upon humans is theoretically beneficial, it does raise significant practical and ethical concerns [14, 55]. Fortunately, other approaches may also be feasible: the impact of both chemical and biological insecticides against *An. arabiensis* can be dramatically enhanced by physically obstructing their exit, rather than their entry, from houses or trap structures, particularly if the active ingredients are applied to the obstructed exit points [94–96]. A promising alternative approach is to provide oral formulations of systemic insecticides to humans to kill mosquitoes that feed upon them [97].

#### Protecting humans against adult mosquitoes when they are active outdoors

**Figure 13:**
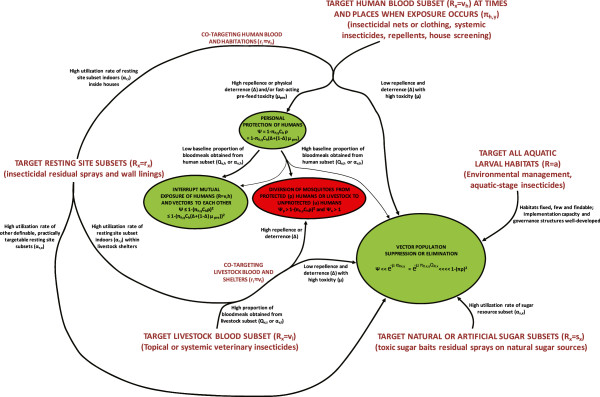
**A schematic representation of how various alternative strategies for targeting vector mosquitoes when they utilize specific resources can suppress (Green) or redistribute, stabilize and even increase (Red) malaria transmission, depending on values for measurable behavioural parameters of the mosquito population and its interaction with interventions** [8, 10, 11, 13–15, 80]**.** Red and green ovals indicate effects upon malaria transmission, with the magnitude of their impact indicated by their size. The relative magnitude of persisting transmission after intervention (*ψ*) is expressed as a function of: (1) the utilization rate (*α*) or probability (*Q*) of targetable subsets (*x*) of a defined resource (*R*, which may be specified as blood (*v*), resting sites (*r*), sugar (*s*) or aquatic larval habitat (*a*)); (2) the coverage of that resource subset (*R*_*x*_) achieved ; (3) the mortality probability (*μ*) of mosquitoes utilizing covered forms of that resource subset; where human blood is the targeted resource, (4) the personal protection (*ρ*) afforded as a result of repellence, irritance or physical deterrence (*Δ*) combined with fast-acting toxicity that occurs before the mosquito can feed (*μ*_*pre*_); and (5) the proportion of exposure that would otherwise occur when that intervention is used . For all parameters described, values approaching or exceeding one are considered high and values approaching zero are considered low. The subscripts *h*, *l* and *i* refer to the subsets *human*, *livestock* and *indoors*.

LLINs and insecticide-treated hammocks may readily be used to protect people sleeping outdoors but are obviously impractical when they are active [84]. The most obvious options for preventing outdoor exposure of humans, especially when they are active and cannot be practically enclosed within a structure like a net, include insecticide-treated clothes [98, 99] and repellents delivered as topical applications or vapour-phase emanators [51, 100]. While such approaches to personal protection of people may achieve valuable community-wide impact upon transmission by simply reducing human-vector contact, they are unlikely to reduce the survival, density or vectorial capacity of the vector population where they obtain most of their blood from animals [8, 10, 11] (Figure 13).

#### Killing adult mosquitoes when they attack livestock

Complementary approaches for killing mosquitoes when they feed upon animals, by treating livestock with topical [101] or systemic insecticides [97] may therefore be invaluable for tackling residual transmission through population control of zoophagic mosquitoes. Note, however, that great care should be taken to ensure the insecticide treatments used have a purely toxic mode of action and lack any irritant or repellent properties that could divert mosquitoes that would otherwise feed on animals to nearby humans [102]. Given that livestock owners primarily apply such veterinary products to protect the animals themselves, any potential for effective malaria vector control will require integration of malaria control priorities into agricultural practices, rather than *vice versa*.

#### Killing adult mosquitoes when they feed upon sugar

An exciting new approach to killing a wide diversity of vector species, regardless of their feeding or resting behaviours, is to treat natural or artificial sugar sources with insecticides [103–105]. The impressive impacts of toxic sugar baits upon three distinct vector species [103–105] are consistent with the high rates at which these mosquito populations utilize sugar [80, 106]. Given the widespread dependence of mosquitoes upon sugar [107–109], especially when infected with malaria parasites [110], it appears that toxic sugar baits may be as generally effective [80] against a wide range of vectors as LLINs are against human-feeding vectors [21] and as insecticide-treated livestock are against animal-feeding vectors [101].

#### Improving implementation systems for larval source management (LSM)

The most direct way to control adult mosquitoes, especially those that are hard to kill because the exhibit various forms of behavioural evasiveness, is simply to prevent them from emerging in the first place. Immature egg, larval and pupal stages cannot fly so they are obviously unable to escape from physical modifications or insecticides applied directly to the aquatic habitats they live in [111]. LSM is perhaps the best established of all mosquito control strategies, with an impressive track record that was largely gained when it was the only mainstream malaria prevention strategy, before the advent of long-lasting adulticides prompted the shift to IRS, and then LLINs, as the highest priority intervention options [112, 113]. Even more encouragingly, renewed investment in developing and evaluating such environmental management or larvicide application methods in Africa have yielded several examples of convincing success [89, 112, 113], leading to revised guidelines for implementation [114]. However, there have also been some examples where impact has been absent, limited or unclear and most successes have come from areas with medium-to-high human population density where aquatic habitats are relatively few, fixed and findable [89, 112–114]. Indeed, seasonally-targeted LSM, implemented only when larval habitats contract to far more manageable levels in the dry season, may have a role to play in the final stages of eliminating malaria transmission [29, 36, 115]. However, it remains difficult to envisage how LSM strategies might be applied routinely and continuously in rural areas with sparse human populations, especially during the wet season peak of transmission when many areas are subject to flooding [112, 114]. Furthermore, even where larval source management is clearly applicable in principle, rigorously evaluated models for effective programme implementation, monitoring, management and governance in contemporary tropical settings [116, 117] remain scarce. Larval source management may have an important role to play in a wide variety of settings, and the implementation systems to deliver it are evolving, but its applicability in rural parts of the tropics will remain limited for the foreseeable future [112, 114] and much remains to be done in terms of defining how to establish and sustain effective programmes based on rigorous, quality-assured entomological surveillance [117–120].

### Learning how to tackle residual transmission with unproven vector control options

There are numerous supplementary vector control options for tackling mosquitoes that persist and mediate residual transmission because they rest outdoors, feed outdoors or feed on animals (Figure 12), and these may be rationally selected based on local surveys of vector behaviours (Figure 13) [8, 28, 80]. However, none of these options have been developed and evaluated sufficiently to justify unreserved recommendation for national-scale roll out by NMCPs. In the absence of an adequate evidence base, NMCPs must either accept the limitations of IRS and LLINs by waiting for the research community to fill remaining knowledge gaps, or they must press ahead as best they can. A medium-to-long term strategy is clearly required to enable NMCPs and their scientific partners to define the needs, markets, ideal product characteristics and optimal delivery systems for such additional control tools through an adaptive learning process [121, 122]. Given the considerable resource constraints that already restrict implementation of LLINs and IRS [123], and the limited evidence available to guide efforts to address residual transmission, perhaps the best way forward for NMCPs is to selectively incorporate supplementary vector control tools into exploratory, pilot-scale integrated vector management programmes [124] that evolve and expand as they establish their own supporting evidence base over the long term, just as the Onchocerciasis Control Programme did [125, 126].

#### Selecting complementary vector control methods by characterizing vector behaviours

While the broad diversity and exciting potential of the options described in Figure 12 is encouraging, this also makes it difficult to select any one of them ahead of another. With limited resources, and a bewildering array of unproven methods for controlling behaviourally evasive vectors to choose from, NMCPs, together with their industrial and scientific partners, need to rationally select a small subset of these options to take forward for concerted programmatic development and evaluation [8, 127]. The likelihood of success or failure of all the options described in Figure 12, are determined by measurable behaviours of mosquitoes and humans (Figure 13). The behavioural determinants of potential applicability and impact of LSM strategies can be reliably assessed with straightforward, well-established field procedures for surveying the distribution of productive habitat among different types of water bodies [112, 114]. In the case of interventions that target blood resources, conceptual frameworks for their selection based on field measurements of vector behaviours, using well-established survey methods that may be readily incorporated into national surveillance platforms (Table 1), are now emerging [8]. This approach may also be extended to a wide variety of other targetable resources that mosquitoes need to survive (Figures 12 and 13), especially if entomological methods for measuring utilization rates for targetable resting sites can be improved [8, 80].

**Table 1:** **A suggested generic plan for strengthening national or regional malaria vector monitoring platforms to incorporate assessment of essential behavioural phenotypes and their influence upon vector control impact, mosquito population dynamics and epidemiological outcomes** [8, 28]

1.	Expand and/or consolidate any existing national network of sentinel surveillance sites for physiological resistance of malaria vector mosquitoes to insecticides [33], ideally integrating these with similar platforms for other common mosquito-borne pathogens, such as lymphatic filariasis. Such sites should also overlap both with existing historical entomological study sites for which baseline legacy data is available, and with national platforms for assessing malaria burden through cross-sectional malaria indicator surveys or quality-assured facility-based surveillance.
2	Establish continuous longitudinal surveillance of mosquito population densities, and the transmission intensity each distinct population mediates, at sites where physiological resistance is monitored, so that the effects of vector control implementation upon seasonal and inter-annual trends can be assessed. Such surveillance platforms are essential to quantify residual transmission and distinguish between the fundamental limitations of an effective vector control strategy delivering incomplete but valuable and sustained impact *versus* an intervention failure, in the strict sense, which allows vector populations and malaria transmission to rebound (Figure 4) [28, 29, 31]. Such continuous, longitudinal surveys of malaria vector population dynamics have never been applied before at nationally representative scales. As such, affordable, practical community- or district-based mosquito trapping schemes, which are nevertheless resourced and managed by centralized national programmes, may need to be developed and evaluated [31, 120]. Given the reliance of scalable trapping schemes, especially those which are community-based, upon widely scattered, field-based personnel who may not always perform adequately [118, 119], it is also essential to establish quality assurance systems in which each of these sentinel sites is regularly and randomly re-surveyed by a centrally coordinated, specialist entomological team using the same trapping methods [31, 120]. Given the diversity of vector species and behaviours across the tropics, setting up such platforms for monitoring mosquito population dynamics may require initial pilot evaluations to select and calibrate suitable trapping methods or validate calibrations from elsewhere.
3.	Incorporate surveys of vector feeding and resting behaviours (using human landing catch by participants protected with drug chemoprophylaxis [128] and backpack aspirator/resting container/screening barrier sampling tools [129–131], respectively) into the quality assurance surveys described above under point 2, so that the extent to which each important vector species feeds on humans, feeds indoors, or rests indoors, can be quantified.
4.	Integrate monitoring of relevant human behaviours [16] and ecology, including resource use and livelihoods, vector control coverage and livestock ownership into national malaria surveys and/or entomologic surveillance platforms, so that their contributions to intervention limitations and failures can be assessed.
5	Where substantial transmission occurs indoors, experimental hut [132–134] facilities should be established at one or two sentinel sites where the most nationally-relevant vector species are abundant, so that the efficacy of vector control interventions can be assessed before and after their introduction [39].

Many countries have now established surveillance platforms for monitoring physiological resistance to insecticides among nationally important vectors at selected sentinel sites [33]. However, the results of these simple insecticide susceptibility assays are not sufficient in themselves for NMCPs to rationally select and implement optimal vector control strategies. This is due to the fact that the small experimental enclosures and insectary rearing conditions they are conducted under are not representative of how wild mosquitoes interact with insecticides in the field. While there are examples of physiological resistance to insecticides resulting in intervention failure [28, 32, 135, 136], LLINs appear to provide valuable levels of personal protection against even highly pyrethroid-resistant vectors [137], and there are examples in which community-level transmission control has been maintained despite such resistance [138]. Several possible reasons for this have been outlined or even demonstrated [139–141]. The quantitative influence of mosquito behaviours upon the successes, limitations and failures of specific intervention strategies, even those as well-established as IRS and LLINs, remain uncertain. It is therefore essential that national entomological surveillance schemes now supplement routine surveys of physiological resistance with regular surveys of mosquito and human behaviours and of underlying resource use and livelihood patterns of those human populations [8, 28, 142]. Platforms for continuous longitudinal monitoring of vector population and malaria transmission dynamics will also be required at the same sentinel locations so that the relevance to programmatic decision-making of any worrying behavioural or physiological traits observed can be directly appraised (Figure 4) [28, 31, 33, 121, 142].

Unfortunately, the examples of well-characterized vector behaviours described in Figures 7 to 10 are merely static stereotypes that fail to capture the considerable variations that occur in behaviour within individual vector species, as a function of time, location and local intervention history. While behavioural variability has been documented for a wide diversity of other important vectors of residual malaria transmission [26, 53, 73, 142], *An. arabiensis* is a particularly good example with which to illustrate this point because it exhibits impressive variability in essentially all its relevant behaviours [28, 59, 142]. The proportion of blood meals it obtains from humans spans the whole range of possible values depending on how it responds opportunistically to fine scale variations in blood host availability [143]. While it feeds outdoors to a considerable degree in parts of Tanzania with high coverage of LLINs [50] or house screening [65, 144], it persists with predominantly indoor feeding habits in parts of neighbouring Kenya with even longer-standing LLIN coverage [46]. Given the opportunity to feed within houses where the occupants lack LLINs, it does so and can be successfully targeted with IRS [93]. However, in stark contrast, it rapidly leaves houses where occupants use LLINs so that supplementary IRS consequently has little impact [39–41].

National and regional malaria control programmes will therefore need to carefully consider how, where and when they monitor important mosquito behaviours [8, 28, 142]. Thus far, even well established entomological methods for measuring vector behaviours have only been applied at village or district scale, and with inconsistent methodology and haphazard distribution across times and locations [2]. This is because they have been predominantly funded through sporadic, short-term research projects. These opportunistic, inconsistent and unreliable sources of vector behaviour measurements now need to be superseded by programmatically-funded, longitudinal monitoring systems operating consistently at national and regional scales (Table 1).

While routine monitoring of mosquito behaviours and population dynamics is essential to characterize and quantify intervention limitations and failures, it can also provide valuable explanatory evidence with which to bolster support for existing interventions like LLINs and IRS [8, 13, 16, 26, 28, 46, 70, 142]. For example, the impressive recent demonstration of the massive impact of LLINs in a holoendemic Senegalese village where residents were provided with almost daily access to testing and treatment [145], is completely compatible with Figures 3, 7, 10 and 11. First, the observed impact on EIR, as measured by human landing catches, appears plausible (Figure 3) based on the expected level of biological coverage that would be achieved for the three vectors present (Figure 10), given that their human-feeding behaviour in this location [66] appears approximately consistent with most other reports for the same species from elsewhere in Africa (Figure 7). Furthermore the EIR values reported after LLIN distribution were measured by fully exposed volunteers so *de facto* transmission levels experienced by protected residents were probably a further order of magnitude lower (Figure 7), reduced from >100 to <1 infectious bite per person per year and therefore consistent with the length of the upper white arrow in Figure 11. Given the ongoing challenge of sustaining funding support for provision of proven interventions like LLINs and IRS [123], it is essential that control programmes can access, interpret and disseminate such data routinely not only to understand and address their own shortcomings, but also to promote and sustain their successes [8, 16, 28, 142].

#### Programmatic evaluation of new intervention options: learning by doing

Where local circumstances allow, NMCPs may incorporate supplementary vector control approaches into exploratory programmes that should include strong monitoring, evaluation and operational research components in the same way that the Onchocerciasis Control Programme did [125, 126], initially through exploratory pilot assessments at manageable, sub-national scales. This strategy will minimize the cost of learning from mistakes along an uncertain route to an adequate evidence base and, eventually, to full-scale implementation. While randomized-controlled trials to evaluate intervention efficacy are of course invaluable contributions to the evidence base, evaluations of effectiveness under non-randomized programmatic conditions are often more relevant, representative and feasible for NMCPs [146].

While such ambitious, NMCP-led programmes for regularly monitoring, evaluating and targeting specific insect behaviours remain to be realized in relation to the vectors of malaria [121, 122], the overwhelming historical success of black fly control by the regional Onchocerciasis Control Programme in West Africa [125, 126] illustrates just how much may be accomplished with a similar strategy of practice-led research, rather than research-led practice. While this approach will undoubtedly take years of troubleshooting, this challenging developmental phase is also an exceptionally useful opportunity for "learning-by-doing". This substantial body of work will probably span at least a decade and represents a historic opportunity to strengthen and institutionalize national expertise through participation in operational research and evaluation at an advanced scientific, rather than merely technical, level. All such investments in these new programmatic monitoring platforms should, therefore, include substantive training components from the outset, especially at postgraduate and post-doctoral level. While a decade may seem like a long time for NMCPs struggling under difficult circumstances to deliver malaria control to huge at-risk populations, it represents the shortest possible period required to develop even a single individual scientist beyond the level of competence to real expertise [147]. Developing relevant expert human resources in the fields of vector biology, epidemiology, informatics, statistics and mathematical modelling will therefore require immediate, concerted and sustained investment in the capacity-strengthening opportunities presented by this extended, but obviously finite, phase of operational research. Crucially, such expert human capacity needs to be established under sustainable and appropriate conditions of ownership and governance at national institutions in malaria endemic countries [121, 122]. While private and para-statal institutions like universities and research institutes have an important role to play, it is the governmental ministries and departments, including the NMCPs themselves, that must receive the highest priority for investing in capacity strengthening [121, 122].

## Abbreviations

EIR: Entomologic inoculation rate
GMEP: Global Malaria Eradication Programme
LLINs: Long-lasting insecticidal nets
LSM: Larval source management
IRS: Indoor residual spraying.
